# WHAT DOES MY PERSONALITY SAYS ABOUT MY FRIENDSHIPS IN LATER LIFE?

**DOI:** 10.1093/geroni/igz038.586

**Published:** 2019-11-08

**Authors:** Emily Lim, Elizabeth A Gallagher, Cindy N Bui, Celeste Beaulieu, Elizabeth Simpson, Laura Driscoll, Jeffrey Burr

**Affiliations:** University of Massachusetts Boston, Boston, Massachusetts, United States

## Abstract

Personality traits, such as those identified in the Big Five Personality Model (i.e., openness to experience, conscientiousness, extraversion, agreeableness, and neuroticism), may be associated with different aspects of friendship among older adults. Additionally, men and women form and maintain their friendships differently, which may result in gender differences in their friendships. This study examined the relationship between specific personality traits and friendship characteristics, including friendship quantity, frequency of social interactions with friends, positive and negative friendship quality. The study also explored whether gender moderates the relationship between personality traits and friendships in later life. This study used data from 7,250 community-dwelling older adults, aged 65 years and above (M=75.4 years old, SD=6.91), who participated in 2012 and 2014 Leave-Behind Questionnaire of the Health and Retirement Study. Results from the linear regression analysis indicated significant main effects for the different personality traits and friendship quantity, quality and social interaction frequency, but no main effect for gender was found. However, the moderating effect of gender was significant for the relationship between specific personality traits (i.e. openness to experience, agreeableness and extraversion) and social interaction with friends, as well as for positive and negative friendship quality. For example, older women who scored high on openness to experience reported significantly lower social contact frequency with friends (B=-.16, p<.05) and higher negative friendship quality (B=.08, p<.05) than men who scored high on openness to experience. Study results provide insights for understanding better how personality traits and gender play a role in friendships in later life.

